# Self-care of hypertension of older adults during COVID-19 lockdown period: a randomized controlled trial

**DOI:** 10.1186/s40885-022-00204-7

**Published:** 2022-07-15

**Authors:** Khitam Alsaqer, Hatice Bebis

**Affiliations:** 1 Public Health Nursing, Cyprus International University, Nicosia, North Cyprus; 2 Public Health Nursing, Eastern Mediterranean University, Famagusta, North Cyprus

**Keywords:** COVID-19, Hypertension, Self-care, Telemedicine, Aged

## Abstract

**Background:**

COVID-19 pandemic has aggravated chronic diseases and health disparities especially hypertension because it is more common among vulnerable populations such as older adults.

**Objective:**

This study aimed to examine the effects of a public health nursing intervention plus m-Health applications for hypertension management on enhancing the self-care, systolic and diastolic of blood pressure, and quality of life in older adults during the lockdown period in Jordan.

**Methods:**

A randomized, controlled trial design was performed in Jordan. A total of 120 participants were randomly allocated to three groups (*n* = 40); interventional group (public health nursing interventions plus m.Health applications) and two control groups (m.Health applications alone group and standard care group).

**Results:**

After 3 months, the interventional group show significantly decreased in systolic blood pressure − 14 (*F* = 16.74, *P* = 0.001), greater improvement in self-care maintenance, monitoring, and confidence (+ 30, + 17.75, + 40.27; *P* < 0.01, respectively) compared to the two control groups. Greater improvement in role limitations due to physical health and due to emotional problems, pain, energy/fatigue, emotional well-being, and social functioning of quality of life (*P* < 0.05) compared to the standard care group. No statistical significant difference was found in diastolic blood pressure (*F* = 3.91, *P* = 0.141), physical functioning (*P* = 0.613), and general quality of life (*P* = 0.060).

**Conclusions:**

This study supports the adoption of technology with nursing intervention as a method of supporting continuity of self-management of chronic illness during the pandemic, and its potential implications for future delivery of health care, not just in Jordan, but across the world.

**Trial registration:**

Clinical Trial.gov (ID NCT04992000). Registered August 12, 2021.

## Background

COVID-19 pandemic has affected all health aspects and aggravated chronic diseases health disparities because it is more common among vulnerable populations such as seniors [1]. Hypertension (HTN) is a long-term chronic disease, affects more than 1 billion people around the world [2]. In Jordan, one-third of Jordanian adults are hypertensive [3], one study expected that the prevalence of HTN may increase 7.2% by 2030 [4], the HTN deaths rate touched 5% of total deaths, ranks Jordan number 7 in the world, and ranks the HTN at number 6 of leading causes of death, after coronary heart disease and stroke, the first and second leading causes of death in Jordan [5]. If high blood pressure is uncontrolled, HTN is the main contributor to heart failure, cardiovascular disease, stroke, kidney disease, and death [2].

Concurrent with COVID-19, care is compromised in the context of lockdown and social distancing. Patients with chronic illness at this time have the risk that they are not obtaining the necessary hospital care, and alternative solutions are required, such as improving the patient’s self-care of his or her chronic disease [6]. Since the hospitals are occupied with COVID-19 cases, the elderly have a perceived threat of COVID-19 and have begun avoiding or delaying health care follow-up [1]. It is essential that we find innovative solutions and sustainable methods for patients with HTN to control their blood pressure (BP), enhance self-care, protect them from COVID-19, and ultimately improve their quality of life (QoL).

Engaging the patient in self-care makes him/her an active participant in the management of illness [7]. Researchers worked to provide patients with the essential knowledge, skills, and abilities to follow treatment recommendations and tolerate BP control [8, 9]. Although they agreed that the best ways to prevent and manage high BP are through reasonable lifestyle changes, i.e., weight loss, low salt diet, stop smoking, limited alcohol, stress management, exercise, and medication [8], this makes the managing of high BP neither more difficult nor easier.

However, to support healthy behaviors, the big electronic revolution provides a good opportunity to involve patients suffering from HTN in the health care process and self-care engagement in a safe space [10, 11]. Moreover, in order to adapt to disruptions during COVID-19, telehealth, mobile health (m-Health), and other technologies which support the self-care process and facilitate access to care are appropriate approaches to protect vulnerable populations who are living with chronic diseases [1, 12, 13]. However, improving the self-care of HTN using m-Health is not a new approach; it has been studied previously by researchers from different disciplines such as technical medicine, family medicine, and pharmacy [14–19], with less attention given to the nursing role.

In literature, especially nursing literature, there is a lack of sufficient scientific research on the effectiveness of m-Health, guided by nurse’s intervention, on self-care of HTN, particularly among older adults [12, 13]. Recently, one study provided a nurse-led program as an example of an effective method of HTN management among older adults [20]. The consequences of the COVID-19 pandemic (isolation, social distancing, and quarantine) show major challenges in the provision of care for older adults with chronic illness [1, 6]. The m-Health offers a great chance for providing care during the lockdown period, which can be applied via mobile apps [21]. Thus, examining m-Health apps guided by public health nursing (PHN) interventions for the management of HTN in older adults during the pandemic can provide important empirical evidence of the effectiveness of such new innovative self-care of HTN interventional methods.

The authors in the present study try to improve and encourage patients who have a chronic disease (e.g., HTN) to be independent in caring for their disease. Self-care is not an alternative for clinic visits; it’s just complimentary for the treatment plan which is not just medication adherence. The authors take advantage of the COVID-19 lockdown situation to be sure that all patients are independent. In this study, we aim to examine three patients outcomes; self-care of HTN, change of systolic BP (SBP), and diastolic BP (DBP), and in three groups of older adult patients with HTN: the interventional group (4-free apps + PHN interventions + education), control group I (4-free apps + education), and control group II (education) during the imposition of lockdown in Jordan as a result of COVID-19 pandemic.

## Methods

### Study design

This study is three-arm groups, single-blind; a randomized controlled trial design was carried out between June and September 2020 in King Abdullah University Hospital (KAUH) in Jordan. KAUH is the largest medical structure in the north of Jordan, serving about 1 million residents [22]. KAUH is a teaching hospital affiliated with Jordan University of Science and Technology. KAUH has an operating capacity of 678 beds, and it can be expanded to 819 beds in emergency situations [23]. In addition, KAUH had received COVID-19 cases in its intensive care unit during the period when the study was conducted.

### Study sample

#### Sample size

G*Power (Heinrich-Heine-Universitat Dusseldorf, Dusseldorf, Germany; http://www.gpower.hhu.de/) was used to calculate the sample size, to achieve a power of 80% at effect size convenient *P* = 0.30, and alpha error probability 0.05; a total sample size of 93 participants was required. Accordingly, for the probability of attrition to follow-up and study withdrawal, the total sample size was estimated to be *n* = 120 for the entire study.

#### Inclusion and exclusion criteria

Participants were enrolled in the study if they were (1) 55 years and above, (2) have follow-up as out-patients of KAUH, (3) had been diagnosed with HTN, (4) on anti-HTN medication—at least one drug, (5) reported that he/she has a personal smartphone (Android)—internet access is not important, and (6) able to read and understand the Arabic language. Participants were excluded if they have Apple smartphones, reported inability to use apps, had any psychiatric or mental illness, had a terminal-stage disease, or were blind or deaf.

#### Recruitment and participants

Once Jordan opened the vital services after the imposition of the lockdown on June 1, 2020, the researcher contacted the medical records department in KAUH and got a list for patients who had an appointment during June (1 June to 30 June) in out-patient clinics. The list included 1500 patients and they were screened to the initial inclusion criteria: age, HTN diagnoses, and free of mental or multiple chronic illnesses as evidences in the electronic system of the hospital.

**Fig. 1:**
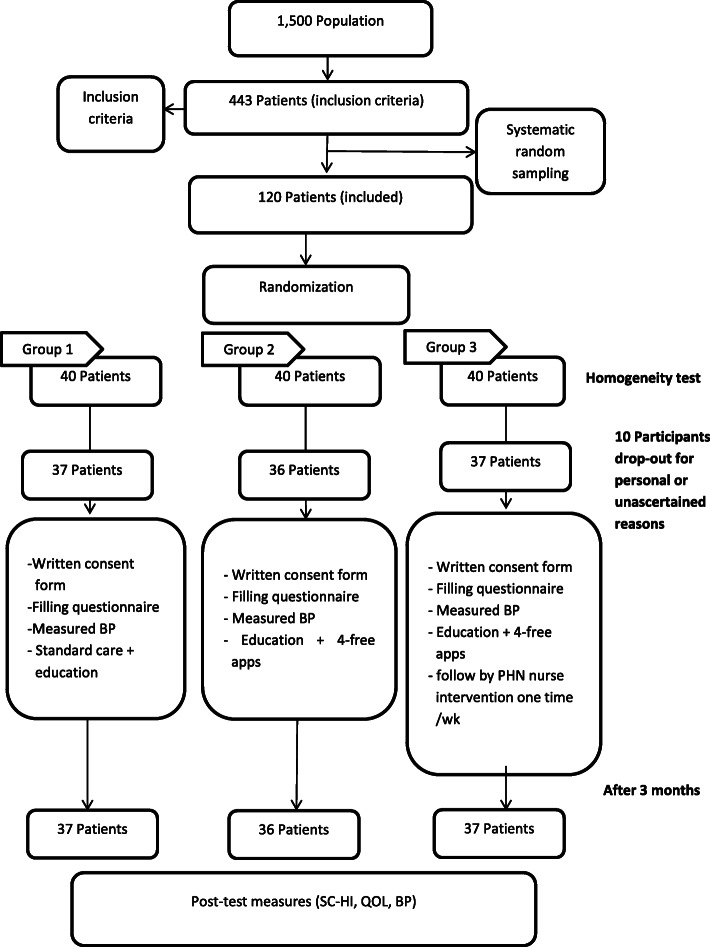
Sample and study process. BP: Blood pressure; PHN: Public health nursing; QoL: Quality of life; SC-HI: Self Care of Hypertension Inventory

Recruited patients (*n* = 443) were asked via telephone if they would participate in the study, after ensuring they could deal with smartphones and met our inclusion criteria. Patients fit for the study aim went through a systematic random sampling to select the sample (*n* = 120). The researcher divided the entire population size by 120 to calculate the sampling interval. The starting point was assigned by the drawing of lots method, and then participants were selected every third interval (Fig. 1).

#### Randomization and blinding

The patients (*n* = 120) were randomly assigned to either intervention group (*n* = 40), apps alone group (*n* = 40), or standard group (*n* = 40) by the simple random sampling method using randomly generated numbers (ratio 1:1:1). Patients and the research coordinator were aware of the groups. However, the principal investigator, co-investigators, and study statistician were blinded.

### The 4-free apps

Free smartphone apps in android were searched through the Play store using keywords such as: self-care, BP, pill reminder, breath, stress management, exercise, and steps. The first 10 pages on the Play store were checked to meet our criteria: free download, English/Arabic language, history availability, and attractiveness in style and colors. The researcher vainly attempted to find an app that included the four main HTN self-care aspects: checking BP, exercise, medication adherence, and stress management. So rather than using one app, the researcher chose one app to help in maintaining and monitoring each one of HTN self-care critical features. While that was not available, the researchers selected one mobile app for each of the four main HTN aspects were (1) *My heart* (BP), (2) *Pill reminder* (medication tracker with alarm), (3) *Breathe easy*, and (4) *StepsApp.*

The 4-free apps were evaluated for content validity, accessibility, usability, and feasibility by focus group interviews and a committee of experts: two nurses with PhDs, two computer technicians, and one cardiologist. They were asked to evaluate the four selected free apps by rating each app from 1 to 4: 1, not relevant; 2, somewhat relevant; 3, quite relevant but needs minor changes; and 4, very relevant. After calculating the content validity index, the 4-free apps were scored ≥0.80 and that was considered acceptable.

### Pilot study

Fifteen patients who are met the study inclusion criteria were enrolled to be in the pilot study. Patients were selected convenes at the intermediate cardiac unit in KAUH, they were interviewed individually in their rooms during hospitalization for 15 min by the researcher, following all COVID-19 protections measures. The researcher presented the education part of self-care of HTN using her personal tap for PowerPoint presentation including pictures. Then, the researcher downloads the four m.apps of self-care of HTN for each participant in his/her phone, saves the 4-apps on quick access mobile screen, and teaches them how to open, follow, and save their data. All patients were evaluated for their understating to education content and acceptance, usability, and feasibility for the 4-selected apps. Almost all patients understand and expressed approval for the 4-free android apps.

### Intervention group

The intervention group was received initial education, 4-free apps, and followed by PHN interventions.

#### Education

Education followed, as per World Health Organization and American Heart Association recommendations for HTN self-care [2]. Subjects: What is high BP; Know your number; How to use a home BP monitor; Choosing a home BP monitor; Changes that patients can make to manage high BP (low salt diet, exercise, medication adherence, stress management, stop smoking, etc.); Educate family members to be part of the BP control process and provide their patient daily reinforcement. Education session presented as well as in the pilot study.

#### The 4-free apps

Participants were instructed to download and use the 4-free apps to facilitate the self-monitoring and detect BP and behavior changes; encourage patients to incorporate them in their life: encourage self-monitoring of BP, and record their readings in the apps’ history through *Myheart-App*, encourage adherence to medication using *Pill reminder-App*, encourage deep breathing exercise as a stress management method using *Breathe easy-App*, and encourage walking and counting steps daily through *Steps-App*. The training session was applied as well as in the pilot study.

#### PHN intervention

Patients were followed via telephone (individual voice calling) for a maximum of 10 min, once weekly; over 3 months by a nurse who is a Ph.D. in PHN and with 7 years’ experience in a cardiac unit. Moreover, the participants continuously can use the chat via a WhatsApp group. The PHN nurse used the three prevention levels (primary, secondary, and tertiary) to guide patients in the self-care process (maintenance, monitoring, and management). The nurse followed the general approaches: (1) assess patient’s maintenance knowledge, attitudes, beliefs, and practices; (2) check the apps’ history for the previous week to detect any changes; (3) and evaluate the patient’s action at that time; (4) finally, give supportive feedback and schedule for next appointment before patient hang up. However, the patients were keeping in touch and sharing their experiences over the three months via the WhatsApp group (e.g., send a screenshot for each app history weekly).

Accordingly, patients were encouraged each other and the PHN nurse provided appropriate interventions based on patients’ needs, which included education, follow-up, screening, counseling, referral, and collaboration with other professionals.

### Apps alone group

Apps alone group received the initial education and the 4-free apps.

### Standard care group

Standard care group received just the initial education.

### Data collections

#### Demographics data

A self-report questionnaire was prepared literally based on such studies which examined such self-care interventions for HTN patients [14, 17, 18, 24, 25]. The 13 questions were on age, sex, marital status, education, economic status, health insurance, smoking, caregiver, job, family history of HTN, health applications experience, duration of diagnosed with HTN, and the number of antihypertensive medication.

#### Changing in systolic and diastolic blood pressure

SBP and DBP were measured and recorded by a clinic nurse who has 9 years’ experience in the cardiac clinic two times for the purpose of the study’s analysis; at baseline and after 3 months following the same assessment process. Patients in setting position with their arms at the chest line, relaxed for 5 min, then measured the BP of the right arm using an electronic sphygmomanometer which was checked for calibrated regularly. BP readings through the 3 months were measured by patients themselves at home.

#### Self-care scale

Self-care of HTN was measured by the Self Care of Hypertension Inventory (SC-HI), which was developed to measure self-care in patients with HTN [26]. SC-HI has the ability to evaluate the effectiveness of such self-care interventions. SC-HI is 23-item in three subscales: measuring self-care maintenance, monitoring, and management, Cronbach’s α were 0.83, 0.75, and 0.83 for the three subscales. For the purpose of this study, authors cross-culturally translated the scale into Arabic in a previous study; Cronbach’s α were 0.82, 0.61, and 0.86 for the three subscales [27]. Each subscale is scored distinctly and identical from 0 to 100, the final score calculated as (the participant score - minimum)/(maximum - minimum) × 100. A separate score of 70 or greater is indicated better self-care.

#### Quality of life scale

QoL of hypertensive patients was measured using the 36-Item Short Form Survey (SF-36). SF-36 questionnaire has been used as a health indicator to screen the health status of persons and evaluate the health interventions [28]. The questionnaire has the feasibility to be administered as a self-report, personal interview, or by telephone, and takes 5–10 min to complete. SD-36 included eight subscales: physical functioning (PF), bodily pain, role limitations due to physical health problems (RF), role limitations due to personal or emotional problems (RE), emotional well-being (EW), social functioning (SF), energy/fatigue (EF), and general health perceptions [28]. SF-36 was frequently used to measure QoL in older adults with HTN; the Arabic version was used in studies with Cronbach’s α ≥ 0.70 [29, 30].

### Data analysis

IBM SPSS ver. 25.0 (IBM Corp., Armonk, NY, USA) was used to perform the statistical analysis. Descriptive statistics were used as mean; standard deviation, minimum, and maximum for continuous variables; frequencies with percentages for categorical variables. Homogeneity of variance at baseline was evaluated using either chi-square or t-test for means differences and frequencies. Normal distribution was tested using the Kolmogorov-Smirnov test; to determine using a parametric or non-parametric test, at 95% confidence interval and a *P*-value of ≥0.05 was considered statistically significant.

Comparisons between the means before and after within group for the three measures of the study were carried out using a paired-samples t-test or Wilcoxon test, a statistical significance was set as P-value < 0.05. Comparison between groups after 3 months was carried out using either one-way repeated measures ANOVA or the Kruskal-Wallis test followed by Tukey multiple comparisons to identify the individual difference.

## Results

### Demographic characteristics

A total of 110 participants who completed the study had 37, 36, and 37 in three groups; 10 participants dropped out from the beginning of the intervention period for personal or unascertained reasons. Patients’ age ranged from 55 to 80 years with a mean age of 60.37 ± 5.60 years, 60.37 ± 5.60 years, and 61.45 ± 7.36 years in each group. Most of the participants were male (56.4%). The majority of participants had a history of HTN in their families (80%). Most had not been using any health application before (97.3%). At baseline, the three groups were statistically no different in their demographic characteristics (*P* > 0.05) (Table 1).

**Table 1:** Characteristics of study participants in the three groups at baseline

Characteristics	Apps + PHN intervention (*n* = 37)	Apps alone (*n* = 36)	Standard care (*n* = 37)	ANOVA/F	*P*-value
Age (yr)	60.37 ± 5.60	60 ± 6.89	61.45 ± 7.36	0.279	0.757
HTN duration (yr)	9.0 ± 8.7	8.3 ± 6.0	7.9 ± 6.4	0.214	0.80
HTN pills (n/day)	1.8 ± 0.8	1.66 ± 0.7	1.8 ± 0.8	0.036	0.96
Sex				1.727^a)^	0.422
Male	21 (56.8)	23 (63.9)	18 (48.6)		
Female	16 (43.2)	13 (36.1)	19 (51.4)		
Marital status				7.519^a)^	0.275
Married	33 (89.2)	35 (97.2)	34 (91.9)		
Single	1 (2.7)	0	0		
Divorce	2 (5.4)	1 (2.8)	0		
Widowed	1 (2.7)	0	3 (8.1)		
Education				10.175^a)^	0.117
Literate	1 (2.7)	2 (5.6)	8 (21.6)		
Primary school	4 (10.8)	6 (16.7)	4 (10.8)		
Secondary school	18 (48.6)	14 (38.9)	16 (43.2)		
University	14 (37.8)	14 (38.9)	9 (24.3)		
Income				8.349^a)^	0.214
Low	13 (35.1)	11 (30.6)	16 (43.2)		
Moderate	14 (37.8)	20 (55.6)	18 (47.3)		
Good	8 (21.6)	5 (13.9)	3 (8.1)		
High	2 (5.4)	0	0		
Health status				3.920^a)^	0.417
Bad	5 (13.5)	3 (8.3)	6 (16.2)		
Not bad	16 (43.2)	23 (63.9)	20 (54.1)		
Good	16 (43.2)	10 (27.8)	11 (29.7)		
Insurance				0.739^a)^	0.691
Yes	34 (91.9)	31 (86.1)	32 (86.5)		
No	3 (8.1)	5 (13.9)	5 (13.5)		
Smoking				0.470^a)^	0.791
Yes	12 (32.4)	11 (29.7)	14 (33.6)		
No	25 (67.6)	25 (69.4)	23 (62.2)		
Social support				3.562^a)^	0.168
Yes	36 (97.3)	36 (100)	34 (91.9)		
No	1 (2.7)	0	3 (8.1)		
Job				1.540^a)^	0.463
Yes	13 (35.1)	8 (22.2)	10 (27)		
No	24 (64.9)	28 (77.8)	27 (73)		
HTN family history				5.777^a)^	0.056
Yes	33 (89.2)	30 (83.3)	25 (67.6)		
No	4 (10.8)	6 (16.7)	12 (32.4)		
Health apps				2.123^a)^	0.346
Yes	1 (2.7)	2 (5.6)	0		
No	36 (97.3)	34 (94.4)	37 (100)		

Data are presented as mean ± standard deviation or number (%)

*PHN* public health nursing, *HTN* hypertension

^a)^Pearson chi-square test

### Changes in blood pressure

At baseline, SBP ranged between 88 and 200 mmHg with a mean of 137.5, 139.5, and 143.2 in each group. DBP ranged between 44 and 111 mmHg with a mean of 81.54, 81.02, and 82.78 in each group. Statistically, show no difference between them (*P* ≥ 0.05).

After 3 months, interventional group and standard group show significant reduction in SBP (− 14, − 7.78; *P* = 0.001 and *P* = 0.003, respectively); no significant decrease in apps alone group (− 5.66, *P* = 0.052). A significant difference was found between the interventional group with standard group and apps alone (*F* = 16.74, *P* = 0.001). Within the three groups, there was no significant change in DBP (*P* ≥ 0.05), as well as no significant changes detected between the three groups (*F* = 3.91, *P* = 0.141) (Table 2).

**Table 2:** Changes in blood pressure of study participants in the three groups at baseline and after 3 months

Blood pressure	Intervention	m.App alone	Standard	Test (ANOVA/Kruskal-Wallis)	*P*-value between groups
SBP					
Before	137.5 ± 22.4	139.5 ± 20	143.2 ± 16.6	0.790	0.456
After	123.5 ± 15.3	133.8 ± 13	135.45 ± 15.6	16.74	0.001*
Mean reduce	–14	−5.66	−7.78		
Paired test	4.47	2.008	3.233		
*P*-value	0.001*	0.052	0.003*		
DBP					
Before	81.54 ± 12.5	81.02 ± 7.2	82.78 ± 9.5	0.745	0.745
After	78.89 ± 8.5	80.63 ± 6.8	82.40 ± 7.90	3.91	0.141
Mean reduce	−2.65	−0.38	−0.37		
Paired test	1.48	0.302	0.325		
*P*-value	0.145	0.764	0.747		

*SBP* systolic blood pressure, *DBP* diastolic blood pressure

* Significant value

#### Self-care (SC-HI)

At baseline, self-care maintenance of study participants ranged between 12 and 72.7 with a mean of 37.06, 36.5, and 33.9 in each group. Self-care monitoring of study participants ranged between 25 and 85 with a mean of 37.06, 36.5, and 33.9 in each group. Self-care confidence of study participants ranged between 22 and 66 with a mean of 37.06, 36.5, and 33.9 in each group. Statistically, show no difference between them (*P* ≥ 0.05).

After 3 months, interventional, apps alone, and standard groups show significant increase in self-care maintenance score (+ 30, + 19.6, and + 10.59, respectively; *P* ≤ 0.05), self-care monitoring score (+ 17.75, + 11.75, and + 1.89, respectively; *P* < 0.05), and self-care confidence score (+ 40.27, + 20.96, and + 0.73, respectively; *P* ≤ 0.05). A significant difference was detected between the interventional group with standard group and apps alone and between the apps alone and standard group (*P* < 0.01) (Table 3).

**Table 3:** Changes in self-care of study participants in the three groups at baseline and after 3 months

SC-HI	Intervention	m.App alone	Standard	Test (ANOVA/ Kruskal-Wallis)	***P***-value between groups
Maintenance					
Before	37.06	36.53	33.93	0.86	0.426
After	67.01	56.13	44.52	48.8	0.001*
Mean reduce	30	19.60	10.59		
Paired test	−16.029	−12.861	−8.08		
*P*-value	0.001*	0.001*	0.001*		
Monitoring					
Before	55.29	54.53	52.70	0.50	0.608
After	73.04	66.33	54.59	37.24	0.001*
Mean reduce	17.75	11.75	1.89		
Paired test	−8.729	−7.197	−2.67		
*P*-value	0.001*	0.001*	0.011*		
Confidence					
Before	41.79	41.88	40.12	0.35	0.703
After	82.06	62.84	40.85	73.08	0.001*
Mean reduce	40.27	20.96	0.73		
Paired test	−16.862	−10.827	−2.36		
*P*-value	0.001*	0.001*	0.023*		

Data are presented as mean values

*SC-HI* Self-Care of Hypertension Inventory

* Significant value

#### Quality of life (SF-36)

At baseline, the means of PF, RP, RE, Pain, EW, SF, and general QoL of the three groups ranged (28.37–63.67) with statistically no difference between them (*P* ≥ 0.05). EF of QoL had mean 44.05, 39.7, and 46.7 in the three groups, showing a statistically significant difference between them (*P* = 0.017). Followed by the Tukey test, the difference was detected between the standard group and the apps alone group (*P* = 0.13).

After 3 months, the interventional group and apps alone group show significant increase in RE (+ 29.28 and + 15.28, respectively; *P* < 0.05), pain (+ 18.38, + 13.19, respectively; *P* ≤ 0.05), EF (+ 11.35, + 10, respectively; *P* ≤ 0.05), EW (+ 9.65, + 6.94, respectively; *P* ≤ 0.05), and SF (+ 14.81, + 8.95, respectively; *P* ≤ 0.05); in the standard group there was no significant difference (*P* ≥ 0.05). Between the three groups there was a significant difference detected between the interventional group and standard care group (*P* ≤ 0.05).

The interventional group and apps alone group show a significant increase in PF (+ 7.84 and + 6.52, respectively; *P* ≤ 0.05) and general QoL (+ 4.32 and + 4.30, respectively; *P* ≤ 0.05), while in the standard group, no significant change (*P* ≥ 0.05). However, between the groups, there was no significant difference (*P* ≥ 0.05).

The interventional group and apps alone group show a significant increase in RF (+ 34.12 and + 24.58, respectively; *P* ≤ 0.05), with no significant change within the standard group (+ 2.02, *P* = 0.324). Between the three groups, a significant difference was detected between the standard care group with interventional group and apps alone (*P* < 0.01) (Table 4).

**Table 4:** Changes in quality of life of study participants in the three groups at baseline and after 3 months

Quality of life	Intervention	m.App alone	Standard	Test (ANOVA/Kruskal-Wallis)	*P*-value between groups
Physical functioning					
Before	45.67	45.41	48.37	0.194	0.824
After	53.51	51.94	48.64	0.492	0.613
Mean change	7.84	6.52	0.27		
Paired test	−4.400	−4.78	−0.52		
*P*-value	0.001*	0.001*	0.600		
Role limitations due to physical health problems					
Before	38.17	35.83	28.37	0.720	0.489
After	72.29	60.41	30.40	20.95	0.001*
Mean change	34.12	24.58	2.02		
Paired test	−5.156	−4.66	−1.00		
*P*-value	0.001*	0.001*	0.324		
Role limitations due to personal or emotional problems					
Before	56.29	62.94	56.74	0.248	0.781
After	85.57	78.23	60.34	10.48	0.005*
t-test	29.28	15.28	3.59		
Paired test	4.642	−3.66	−0.99		
*P*-value	0.001*	0.001*	0.325		
Pain					
Before	47.43	49.72	53.58	1.421	0.246
After	65.81	62.91	55.25	10.38	0.006*
Mean reduce	18.38	13.19	1.67		
Paired test	−7.195	−7.50	−1.43		
*P*-value	0.001*	0.001*	0.161		
Energy/fatigue					
Before	44.05	39.79	46.75	4.232	0.017
After	55.40	49.79	47.16	13.52	0.001*
Mean reduce	11.35	10.00	0.40		
Paired test	−7.999	−9.44	−0.44		
*P*-value	0.001*	0.001*	0.661		
Emotional well-being					
Before	60.21	61.44	63.67	1.154	0.319
After	69.86	68.38	64.64	5.92	0.052
Mean reduce	9.65	6.94	0.97		
Paired test	−5.715	−6.36	−1.42		
*P*-value	0.001*	0.001*	0.163		
Social functioning					
Before	51.00	50.76	53.71	0.400	0.671
After	65.81	59.72	54.39	9.70	0.008*
Mean reduce	14.81	8.95	0.67		
Paired test	−7.346	−6.14	−0.46		
*P*-value	0.001*	0.001*	0.644		
General					
Before	56.08	55.00	55.94	0.159	0.853
After	60.40	59.30	55.94	5.61	0.060
Mean reduce	4.32	4.30	0.00		
Paired test	−4.852	−5.74	0.00		
*P*-value	0.001*	0.001*	1.000		

Data are presented as mean values

* Significant value

## Discussion

This study took place during the imposed lockdown period in Jordan as a result of the COVID-19 pandemic to provide empirical evidence of using the mobile apps with PHN intervention, in order to improve HTN self-care among older adults at a safe distance. This randomized controlled trial, the two-blind design achieved a significant reduction in SBP, better self-report of maintenance, monitoring, and confidence self-care, improvement in the RP, RE, Pain, EF, EW, and SF of QoL over 3 months of 4-free apps with PHN intervention compared to standard care and using the 4-free apps alone.

Nurses play an important clinical role to complete the picture of management away from the hospital setting and nurse-led interventions have proved effective to improve controlling of BP and healthy outcomes in hypertensive patients [31]. Self-care of HTN was measured in a different way in literature: knowledge of BP management, self-care behavior, and the majority was focused on measuring medication adherence [18, 20, 25, 31, 32]. This study uses a comprehensive instrument to measure the self-care of HTN among older adults [26, 27]. At baseline, the participants showed low maintenance and confidence (33.9–37 and 40–41.8, respectively) and moderate monitoring self-care (52.7–55.2).

**Fig. 2:**
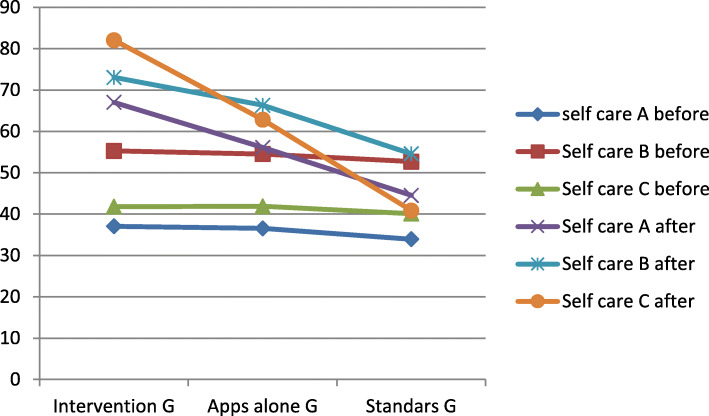
Before and after changes of Self-care of Hypertension Inventory items mean. A: maintenance, B: monitoring, C: confidence

After the 3 months, a significant improvement was detected in self-care maintenance, management, and confidence among the intervention group (+ 30, + 17.7, + 40.2; *P* < 0.001) and better than the two control groups (*P* < 0.001) (Fig. 2); in a study that used the m-Health to improve the self-care of heart failure, the significant improvement was found in management and confident self-care (+ 8.7, + 7.03; *P* < 0.001) while no significance found in the improvement of maintenance self-care (+ 5.4, *P* = 0.93) [32]. Although, different studies, as well as our two control groups (standard care and apps alone), have a significant improvement in the self-care (*P* < 0.05) [18, 33], the interventional group (apps + PHN intervention) had significantly better improvement than apps alone and standard groups (*P* < 0.05). Accordingly, the 4-free apps plus PHN intervention seems to have a better impact on the patient’s self-care level. Hence, the measures of engagement with the technology are a critical portion of self-monitoring, future research will include this component in the analysis within the context of COVD-19 restrictions.

However, nurse-led studies found a significantly greater improvement in self-care behaviors and satisfaction with hypertensive care in their interventional group than the control group [18, 25]. Two studies could not find any significant improvement in the self-care behaviors or adherence to medication even when using the m-Health as self-monitoring [14, 17]. This can be related to the period after the intervention; 6 months may have the potential of the unsustainability of self-care. In combining the apps with PHN intervention, there is a high chance in maintained, monitored, and confident self-care of patients with uncontrolled BP at the community level.

The QoL remained consistent among groups utilizing m-Health intervention to improve the HTN management in an underserved community [19]. In this study, while there is a significant difference between the intervention group and the standard care group in the RP, RE, Pain, EF, EW, and SF of QoL (*P* < 0.05), we found no significant difference between them in the PF, EW, and general QoL (*P* ≥ 0.05). In literature, the physical component and mental component of QoL were significantly enhanced in the interventional group that addressed a nurse-led HTN management program among older adults’ patients [20]. On another side, studies that addressed nurse-led management was no statistically significant difference detected in the QoL between the two groups after the intervention [25]. We can explain our results to the restriction measures of the COVID-19 pandemic (e.g., social distance, social isolation, and curfew) which may limit the physical function and force the participants to stay at home. However, we try to pass this condition by encouraging them to perform a home exercise. Thus, maybe the period of 3 months in this study is insufficient to detect the difference in the QoL measures.

COVID-19 pandemic and its consequences are associated with mental health impacts mostly among older adults, lockdown restrictions, and keeping social distance leading to high risk of loneliness which can lead to anxiety and depression far ahead [34, 35]. Accordingly, some studies suggested that performing such daily physical activity during the lockdown period may improve some of the undesirable mental health impacts that older adults experience while curfew and obeying the protection measures during the COVID-19 pandemic [36]. In our study, we give this opportunity for patients to engage in some individual exercise at home-like walking in the yard and performing a deep breathing exercise. Health promotion directed at embracing and maintaining confident health-related activities must be utilized to address mental distress during the pandemic.

**Fig. 3:**
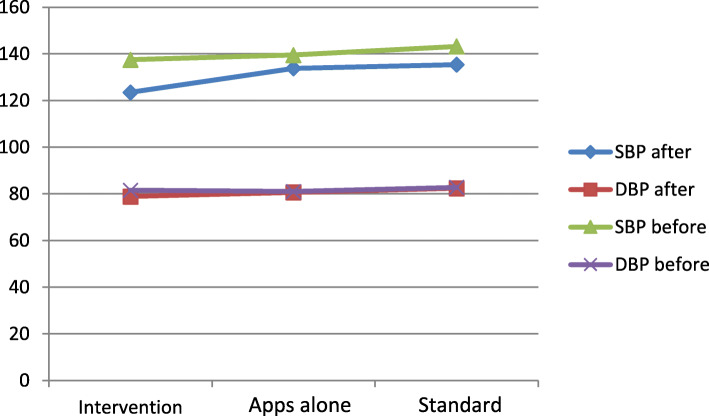
Before and after changes of systolic blood pressure (SBP) and diastolic BP (DBP) means

According to a meta-analysis, studies that used the m-Health had a better BP reduction in intervention groups; SBP (− 3.78 mmHg) and DBP (− 1.57 mmHg) compared with control groups (*P* < 0.001) [13]. In this study, the baseline of SBP mean was consent with other studies [14, 19, 20, 37] that carried out the HTN management (137 − 143 mmHg) and provide a significant reduction in SBP (− 15; 95% CI, *P* < 0.001) after the 4-free apps plus the PHN intervention. Moreover, the interventional group shows much greater reduction than the two control groups (*P* = 0.001) (Fig. 3), while studies that examined different interventions to improve BP achieved a reduction in the SBP − 2.7, − 8.3, − 16, − 7.8 [14, 19, 20, 37]. The highest reduction (− 16) was in a study that used telehealth counseling by a nurse giving priority to the role of PHN in the potential for greater enhancing the self-care of HTN, especially in such crisis situations which preclude the continuity of health services. Participants might have been anxious and worried about COVID-19; this may raise the suspicion that the participants were more committed and engaged seriously. In essence, when patients feel danger, they become more determined.

DBP in this study was (81–82.7 mmHg) coinciding with other studies’ mean (84–90) [12, 14–17]. However, we found no significant reduction in the DBP either in an interventional group or two control groups (− 2.65, − 0.38, − 0.37; *P* > 0.05), as well as no significant difference detected between the groups (*F* = 3.91, *P* = 0.141) (Fig. 2). This result coincides with those studies with different interventions that were no significant reduction found in DBP (− 2.7, − 3.5, − 5.7, − 2.0; *P* > 0.05) [14, 19, 20, 37]. Thus, the clinical effects in patients with uncontrolled BP could be noted better in SBP than DBP and more time is needed to detect the significant changes in DBP.

Limitations of this study were choosing four apps instead of one app, which will increase the burden on patients to log into four different apps. Three months were not enough to detect changes of QoL. Researchers recommend for future study to develop one app which includes the main aspects of HTN self-care and to follow participants for 6 months. Further statistical analysis needed to detect the effects of patients’ characteristics and use more variables that may reflect the behaviors changes (i.e., weight, or the body mass index).

## Conclusions

In this study, with aggregate disease burden from HTN globally, COVID-19 offers the opportunity for the public health nurse to muster and put into action the potentially effective integrated m-Health for self-care and control of BP plus nursing interventions, which provide an efficient approach for managing large numbers of hypertensive patients in a community setting during a national lockdown.

Our findings indicate that the combination of technical and nursing interventions may be effective for the promotion of QoL and HTN self-care, resulting in a statistically and a clinically significant decrease in BP among older adults with HTN, compared to using technology alone or receiving standard care.

This study points to the adoption of technology with nursing intervention as a method of supporting the continuity of self-management of chronic illness during the pandemic, and its potential implications for future delivery of health care, not just in Jordan, but across the world. It is strongly predicted that we will face local and global problems that will affect health services in the future. We recommend repeating the study intervention with different groups and with follow-up studies.

## Data Availability

The data that support the findings of this study are available from the corresponding author, upon reasonable request.

## Abbreviations

BP: Blood pressure
DBP: Diastolic of blood pressure
EF: Energy/fatigue
EW: Emotional well-being
HTN: Hypertension
KAUH: King Abdullah University Hospital
m-Health: Mobile health
PF: Physical functioning
PHN: Public health nursing
QoL: Quality of life
RCT: Randomized controlled trial
RE: Role limitations due to personal or emotional problems
RF: Role limitations due to physical health problems
SBP: Systolic blood pressure
SC-HI: Self Care of Hypertension Inventory
SF-36: Short Form Survey
SF: Social functioning
